# A Cerebrovascular Accident Presenting With Bilateral Vocal Cord Paresis

**DOI:** 10.7759/cureus.17840

**Published:** 2021-09-09

**Authors:** Kyle Wiseman, Dhairya Gor, Gautham Upadrasta, Ndausung Udongwo, Kara Lanpher, Steven Douedi, Swapnil V Patel

**Affiliations:** 1 Internal Medicine, Jersey Shore University Medical Center, Neptune City, USA

**Keywords:** cerebrovascular accident (stroke), stridor, cerebellar hemorrhage, vocal cord paresis, bilateral vocal cord paresis, vocal cord paralysis, bilateral vocal cord paralysis, stroke

## Abstract

Diagnosing a stroke requires careful attention to clinical indicators on physical exam, especially the more subtle manifestations of cerebellar lesions. An 85-year-old male with vascular risk factors and new-onset atrial fibrillation was admitted for left upper extremity weakness, headaches, and tremors. The patient developed stridor during hospitalization and was found to have a new cerebellar infarct with hemorrhagic transformation on computed tomography (CT) of the head, with laryngoscopy showing bilateral vocal cord paresis. While strokes outside of the cerebellum are a known cause of unilateral vocal cord paresis, cerebellar strokes are a rare culprit and rarely cause bilateral cord paresis. Consideration beyond the more common pulmonary and iatrogenic causes of vocal cord paresis should be considered, with particular attention to stroke.

## Introduction

Cerebrovascular accidents (CVA) in the cerebellum usually present with dizziness, nausea, vomiting, gait disturbances, and headache [1]. Neurological signs can include dysmetria, dysarthria, nystagmus, and ataxia [1]. We present a case of cerebellar infarction with hemorrhagic conversion presenting with stridor due to a bilateral vocal cord paresis. To our knowledge, vocal cord paresis is an atypical presentation of cerebellar infarction.

## Case presentation

An 85-year-old male with a medical history of hypertension, hyperlipidemia, benign prostatic hyperplasia, stage III chronic kidney disease, and vasovagal syncope was brought to the emergency department (ED) and complained of left upper extremity shaking and an abrupt, non-localized headache that started a day prior to admission associated with tinnitus, vision changes, generalized weakness, nausea, vomiting, and new left upper extremity weakness. There was no report of loss of consciousness, incontinence, or tongue biting. Family history was remarkable for coronary artery disease in his father. He smoked two packs of cigarettes per day for about 32 years. His daily home medications were aspirin 81 milligrams (mg), atorvastatin 40 mg, lisinopril - hydrochlorothiazide 20 - 25 mg, atenolol 25 mg, and finasteride 5 mg. On arrival, his Glasgow Coma Scale (GCS) score was 15 (E 4, V 5, and M 6), and his NIH Stroke Scale/Score (NIHSS) was 2 (1 each for limb ataxia and dysarthria). Initial vitals were a blood pressure of 174/83 mmHg, heart rate of 151 beats per minute, respiratory rate of 18 breaths per minute, temperature of 98.4℉, and oxygen saturation (SpO_2_) of 98% on room air. On physical examination, he was alert and oriented to name, place, and time. Cranial nerves II to XII were intact. Strength was 5/5 in the upper and lower extremities. Sensations were intact, with no dysmetria noted. The cardiopulmonary examination was only remarkable for an irregular heartbeat. Other examination findings were noncontributory. The laboratory results on admission are presented in Table 1.

**Table 1:** Initial laboratory results

Laboratory Test	Results	Reference Range
Hemoglobin (g/dL)	14.4	12.0-16
White blood cell count (10^3^/µL)	11.8	4.5-11.0
Sodium (mmol/L)	139	135-146
Potassium (mmol/L)	3.9	3.5-5.0
Glucose (mg/dL)	146	70-110
Blood urea nitrogen (mg/dL)	31	7.0-18.0
Creatinine (mg/dL)	1.51	0.44-1.0
Low density lipoprotein (LDL) (mg/dL)	96	<100
Aspartate aminotransferase (U/L)	29	10-42
Alanine aminotransferase (U/L)	37	10-60
Total bilirubin (mg/dL)	1.9	0.2-1.2

Electrocardiogram confirmed new-onset atrial fibrillation with a rate of 132 beats per minute with no ST or T-wave changes. A non-contrast computed tomography (CT) scan of the head was negative for cerebral hemorrhage and showed no signs of large vessel territory infarct. He was not a candidate for tissue plasminogen activator (tPA) due to the delayed onset of symptoms. He was started on diltiazem and anticoagulated with low-intensity heparin drips for new-onset atrial fibrillation. CT angiogram (CTA) of the head and neck revealed 50% to 60% stenosis at the right proximal internal carotid artery, 30% stenosis at the left proximal internal carotid artery, and mild stenosis at the origin of the right/left vertebral artery. Echocardiogram was remarkable for an ejection fraction of 55%-60% with mild mitral regurgitation.

**Figure 1:**
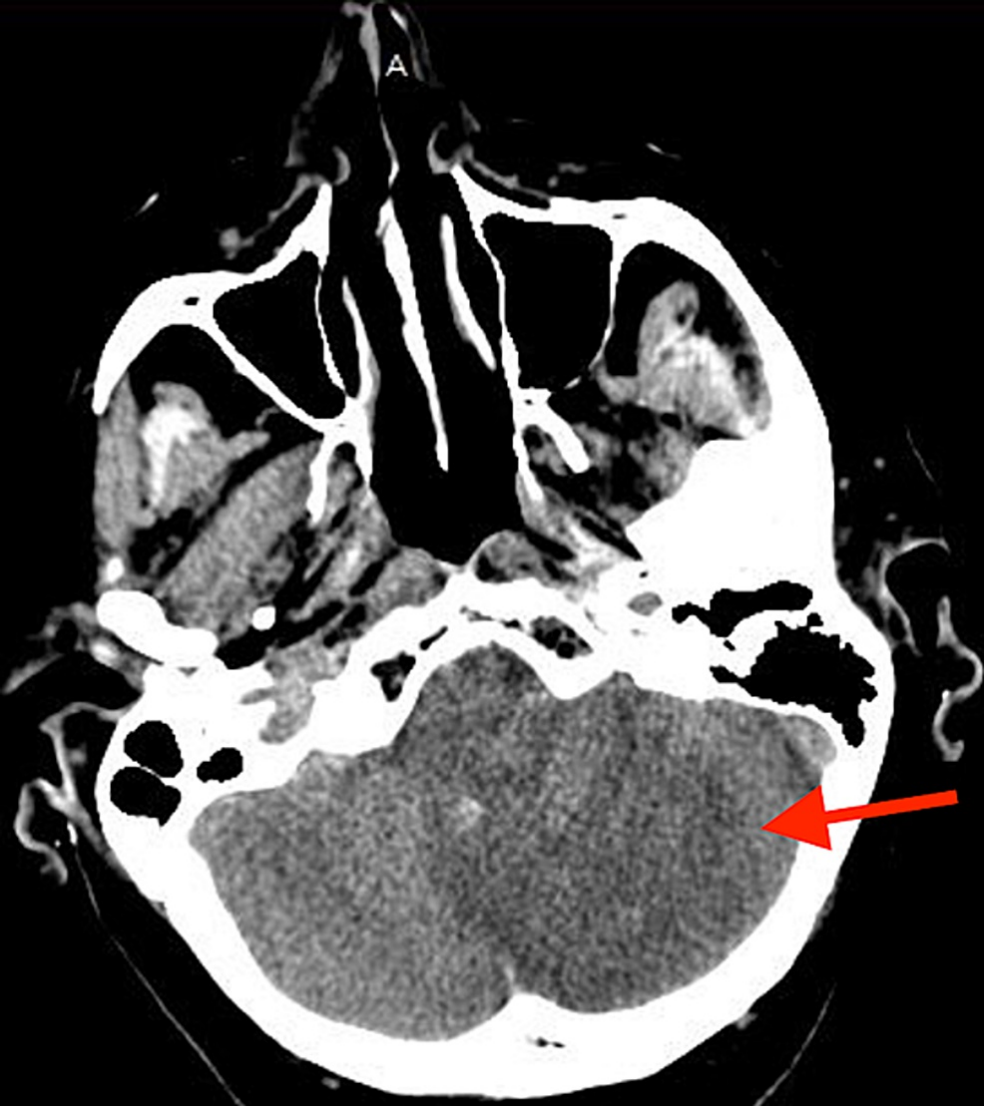
Computed tomography scan of the head without contrast showing acute infarct of the left cerebellar hemisphere with the hemorrhagic conversion with effacement of the fourth ventricle.

On the second day of hospitalization, the patient developed hypoxia, new-onset stridor, persistent coughing spells, shortness of breath, dysmetria on the finger to nose testing (left more than right), and altered mental status. On assessment, there was expressive aphasia, left upper extremity weakness (3/5), and disorientation noted. He received racemic epinephrine, 125 mg solumedrol, and helical oxygen 4L due to concern for an allergic reaction. A repeat CT scan of the head revealed an acute infarct in the left inferior to medial cerebellar hemisphere with mild associated hemorrhagic conversion. Associated mass effects, including compression of the fourth ventricle and mild left cerebellar tonsillar herniation, was also noted (Figure 1). Protamine sulfate was administered and heparin/aspirin was discontinued. Neurosurgery was consulted and a decision was made with the patient and family to not pursue any surgical interventions due to the risks. Bedside laryngoscopy done by an otolaryngologist revealed partial limitation of bilateral vocal cord abduction on inspiration, possibly due to cerebellar hemorrhage and mass effect on cranial nerve X. The patient was transferred to the neurointensive care unit (ICU) and his cerebral edema was treated with 3% hypertonic saline. The patient never required intubation and an MRI was not completed due to patient refusal.

**Figure 2:**
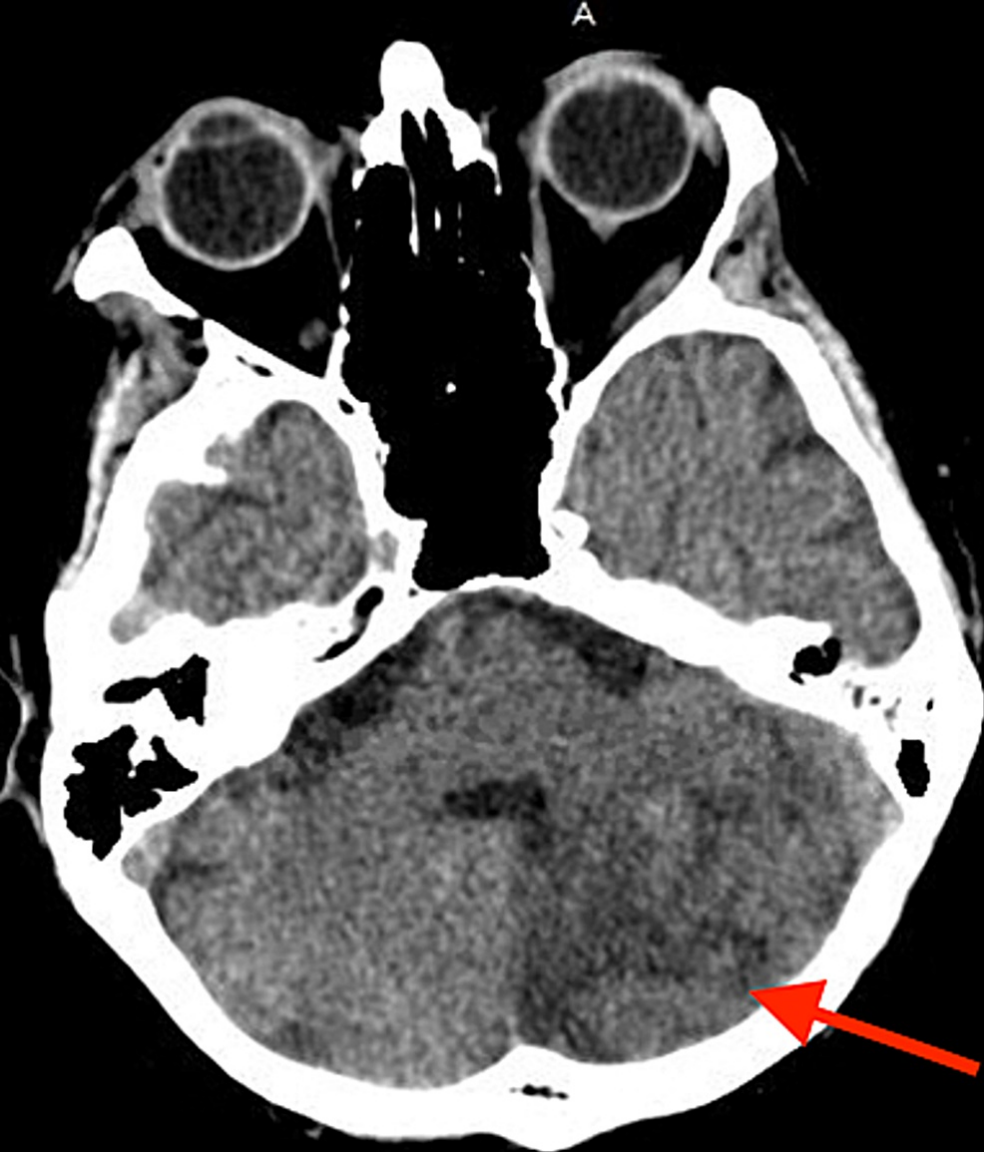
Computed tomography scan of the head without contrast showing an evolution of the left cerebellar infarct with patchy low attenuation in the left cerebellum that appears slightly smaller compared to prior CT.

The patient improved over one week and was discharged to a rehabilitation center. Twenty-three days later, a repeat CT scan of the head showed an evolution of the left cerebellar infarct with patchy low attenuation of cerebellar hemorrhage that appeared slightly smaller when compared to prior imaging (Figure 2). On an outpatient follow-up visit three weeks later, his stridor had significantly improved.

## Discussion

The cerebellum is the main component of the hindbrain and is located behind the fourth ventricle, pons, and medulla in the posterior cranial fossa [1]. The cerebellum is divided from the cerebrum by the tentorium cerebelli, which is an extension of the dura mater [1,2]. The vermis connects the cerebellum's two hemispheres and there are three cerebellar lobes: anterior, posterior, and flocculonodular [1,2]. While the cerebellum is smaller than the cerebrum, it is more neuron-dense, containing around 80% of the brain's neurons [2]. The cerebellum attaches to the brainstem via three groups of nerve fibers: the superior, middle, and inferior cerebellar peduncles, which have efferent and afferent fibers connecting with the remainder of the nervous system [2]. Regarding vasculature, the superior cerebellar artery (SCA), the anterior inferior cerebellar artery (AICA), and the posterior inferior cerebellar artery (PICA) all emerge from the posterior circulation and are engaged in cerebellar vascular supply [1,2]. In our case, CTA of the head and neck showed moderate proximal ICA and right vertebral artery stenosis. Given the proximal nature of the patient’s stenoses and the collateral circulation from the Circle of Willis, there were fewer classic cerebellar symptoms, which is consistent with the typical non-focal findings seen with cerebellar infarcts compared to the more focal findings with anterior circulation infarcts [3].

Cerebellar strokes can manifest in a variety of ways, including headaches, disorientation, paresthesias, and dizziness [3,4]. The diagnostic modality for cerebellar infarction is magnetic resonance imaging (MRI) [1]. Many cerebellar infarctions may only be discovered using MRI due to a lack of clinical signs and low sensitivity of CT scans [1]. We were unable to obtain an MRI since the patient was disoriented at the time of consent and the family later declined.

Several risk factors have been identified for strokes in general, including modifiable and non-modifiable categories [5]. Hypertension, diabetes, hyperlipidemia, smoking, alcohol, and cardiac disease are modifiable risk factors, while age, gender, and ethnicity are some of the non-modifiable risk factors [4-6]. Hypertension is the most significant contributor to stroke, albeit its role varies depending on the subtype [5]. Ischemic strokes account for the bulk of cases, with small vessel arteriosclerosis, cardioembolism, and large artery atherothromboembolism being the most common causes [5].

Around 10% of all intracranial hemorrhages are located in the cerebellum [7]. The limited space of the posterior fossa allows for the mass effect to easily occur [7]. Cerebellar hemorrhages often occur in the dentate nucleus, however, can also occur near the cerebellar peduncles [8]. Our patient’s hemorrhage possibly had a mass effect on the cerebellar peduncles, affecting the pathway of special visceral efferent nerve fibers originating from the nuclear ambiguous that join the vagus nerve through the jugular foramen and play a role in abduction/adduction of laryngeal muscles [8,9]. This would present as stridor; but unlike in our patient, hypoxia and additional morbidities can also occur in the more severe cases [8].

The majority of strokes affecting the vocal cords are not located in the cerebellum [8]. This is evidenced by a 1999 study that showed vocal cord paralysis in 20.4% of the studied patients who presented with various ischemic strokes, with only one of the cases demonstrating a cerebellar infarct. Additionally, all of the cases included in the study presented with unilateral rather than bilateral vocal cord paralysis, further highlighting the unique nature of our case presentation [8].

Bilateral vocal cord paresis/paralysis, in general, is usually seen in conjunction with central nervous system pathologies such as tumors, multiple sclerosis, and rarely CVA [10]. In 2014, a similarly unique case was reported in which a patient with a history of right medial medullary infarct who presented with inspiratory stridor was found to have bilaterally abducted vocal cords on laryngoscopic evaluation [11]. The patient’s hospital course was complicated by the onset of right-sided “heaviness” and dysphagia. Imaging confirmed a subacute infarct in the left paramedian pons [11]. This 2014 case report is an example of a similar presentation to our case given the bilateral vocal paralysis, but differing in that the stroke was located outside the cerebellum.

Our patient’s presentation is unique in that a direct neurological pathology caused a bilateral vocal cord paresis presenting as stridor. Most causes of stridor are pulmonary conditions like tracheal compression or aspiration [12]. Additionally, vocal cord paresis and paralysis are usually related to pathology in the peripheral nervous system such as direct damage to the recurrent laryngeal nerve post-thyroid surgery [12]. Thus when a patient with risk factors for stroke develops stridor, consideration toward an insidious central nervous system pathology such as cerebellar hemorrhage should be given in addition to the common pulmonary and peripheral nervous system causes.

## Conclusions

Unilateral vocal cord paresis is commonly seen in cerebral strokes. Though often attributed to a pulmonary or peripheral nervous system pathology, stridor can indeed be the sign of a more insidious process due to vocal cord paresis from a cerebellar stroke with hemorrhagic conversion, leading instead to a bilateral vocal cord paresis. A broad differential diagnosis that includes CNS pathologies in a patient with vascular risk factors who develops stridor should always be entertained.

## References

[1] De Cocker LJ, Lövblad KO, Hendrikse J (2017). MRI of cerebellar infarction. Eur Neurol.

[2] Jimsheleishvili S, Dididze M (2021). Neuroanatomy, Cerebellum. https://www.ncbi.nlm.nih.gov/books/NBK538167/.

[3] Compter A, Kappelle LJ, Algra A, van der Worp HB (2013). Nonfocal symptoms are more frequent in patients with vertebral artery than carotid artery stenosis. Cerebrovasc Dis.

[4] Berry DC, Rafferty A, Tiu K, Platt-Mills TF (2017). Cerebellar stroke: a missed diagnosis. Adv Emerg Nurs J.

[5] Murphy SJ, Werring DJ (2020). Stroke: causes and clinical features. Medicine (Abingdon).

[6] Rojsanga W, Sawanyawisuth K, Chotmongkol V, Tiamkao S, Kongbonkiat K, Kasemsap N (2019). Clinical risk factors predictive of thrombotic stroke with large cerebral infarction. Neurol Int.

[7] Datar S, Rabinstein AA (2014). Cerebellar hemorrhage. Neurol Clin.

[8] Venketasubramanian N, Seshadri R, Chee N (1999). Vocal cord paresis in acute ischemic stroke. Cerebrovasc Dis.

[9] Soriano RM, Gupta V (2021). Anatomy, Head and Neck, Larynx Nerves. https://www.ncbi.nlm.nih.gov/books/NBK557742/.

[10] Salik I, Winters R (2021). Bilateral Vocal Cord Paralysis. https://www.ncbi.nlm.nih.gov/books/NBK560852/.

[11] Allam H, Kassar D, Al Khalili Y, Karadaghy A, Alshekhlee A (2015). Brainstem ischemia presenting with inspiratory stridor. Neurol Clin Neurosci.

[12] Park T, Kim Y, Ko DH, McCullough G (2010). Initiation and duration of laryngeal closure during the pharyngeal swallow in post-stroke patients. Dysphagia.

